# Abdominal cocoon syndrome, a case report of a rare disease entity causing intestinal obstruction

**DOI:** 10.1016/j.ijscr.2021.106401

**Published:** 2021-09-13

**Authors:** Farah Mohammed, Mohamed Abdulkarim, Ammar Ibn Yasir, Osman Taleballah, Dafalla Shani, Nadir Salih

**Affiliations:** a MBBS, National Ribat University, Sudan; b Alzaiem Alazhari University – Department of Surgery, Sudan

**Keywords:** Sudan, Cocoon, Sclerosing, Encapsulating, Peritonitis, Case report

## Abstract

**Introduction and importance:**

Abdominal cocoon syndrome is a rare condition characterized by small bowel encapsulation by a fibrous membrane or a cocoon-like sac. It is an uncommon cause of intestinal obstruction. Less than 300 cases have been reported from all over the world. This is the first case of such a disease entity to be reported from Sudan.

**Case presentation:**

A young female patient, presented with features of intestinal obstruction that was managed conservatively. Failure of the conservative management has warranted a laparotomy.

**Clinical findings and investigations:**

Her features were suggestive of intestinal obstruction that was confirmed radiologically.

**Interventions and outcome:**

Laparotomy revealed a membrane-like fibrous material and extensive multiple loops adhesions, findings consistent with primary sclerosing encapsulating peritonitis (PSEP), also known as abdominal cocoon's disease. The membrane was excised and adhesiolysis was done. Intestinal obstruction was relieved after surgery and the patient showed good outcome.

**Conclusions:**

Abdominal cocoon syndrome is a rare cause of intestinal obstruction.

**Relevance and impact:**

The takeaway lesson from this case would be that the PSEP should be sought in any patient with no clear cause for obstruction can be identified. A contrast-enhanced CT scan is the diagnostic modality of choice. Finally, we think that the disease is underreported from Africa and more efforts should be carried out to increase patients' access to healthcare especially in rural areas with no access to hospitals in order to bring more cases to light. This case report has been reported in line with the SCARE Criteria (Agha et al., 2020 [17]).

## 1 Introduction

### 1.1 Background

Primary sclerosing encapsulating peritonitis (PSEP) is a disease characterized by small bowel matted together by a fibrous membrane or a cocoon-like sac [1]. It is an uncommon cause of intestinal obstruction. It is more common in tropical and subtropical areas with young adolescent females being the most affected age group.

### 1.2 Rationale

What makes this case unique among the reported cases of such a condition is that this is the first case to be reported from Sudan and one of the very few cases to be reported from Africa [1]. With only 6 cases reported from this continent and given the fact that the disease is common in tropical and subtropical areas, we think that PSEP prevalence is no less common than in other parts of the world with a similar description, and the disease is underreported in this part of the world.

### 1.3 Guidelines and literature

Sclerosing encapsulating peritonitis (SEP) is a rare chronic inflammatory condition that commonly presents as intestinal obstruction, either on-and-off or constant depending on severity. This condition can be classified into primary or secondary, in terms of etiology [2]. This condition is usually diagnosed intra-operatively due to the difficulty of the diagnosis via laboratory and imaging modalities [3]. There are no clear guidelines regarding the management of SEP. However, surgical intervention including excision of the sac, adhesiolysis, and prophylactic appendectomy gives a good outcome [1]. This case report has been reported in line with the SCARE Criteria (Agha et al., 2020 [17]).

## 2 Patient information

A 16-year-old female student, presented to our emergency department by her family with complaints of persistent vomiting, nausea, constipation, and epigastric abdominal pain along with weight loss. She has a history of on-and-off constipation, but no previous history of hospitalization or surgical operations. She has no history of allergies or chronic medications consumption. She lives with her parents and does not smoke or drink alcohol. Regarding her family history, there was no history similar presentation.

## 3 Clinical findings

Her physical examination showed a distended abdomen with full flanks, and it was dull on percussion. There was tenderness over the epigastric area but no grading. There was mild hepatomegaly, and she was slightly jaundiced. Her vital signs were as follows: PR = 78, BP = 80/70, T = 36,9 and SPO2 = 98% on room air. Her respiratory and cardiovascular systems were normal.

## 4 Timeline

Our patient has presented first to the emergency department with constipation and vomiting and was admitted to the internal medicine department. Three days after admission and conservative treatment with no response, we have been called in the surgical department for consultation. Based on her symptoms, a diagnosis of bowel obstruction was made and emergency surgery was arranged which revealed the abovementioned findings. She was discharged one week later in a good condition. A two-week later follow-up visit was scheduled which was satisfactory with a good outcome. A one-month later, phone interview was scheduled given the fact that the patient has returned to her home country (South Sudan).

## 5 Diagnostic assessment and interpretation

### 5.1 Investigations

Lab investigations revealed a normal WBCs count with low Hb at level of 8.5. Regarding her electrolytes, serum calcium and potassium were low (6.2 mg/dL, 2.03 mmol/l, respectively) (normal values Ca+ from 8.5 to 10.5, K+ from 3.5 to 5.3). Renal function tests and bleeding profile were normal, while her liver function tests including total protein, albumin and T bilirubin were low (5.41 g/dl, 2.97 g/dl, 0.605 g/dl, respectively) (normal values total protein from 6.4 to 8.3 g/dl, albumin3.5 to 5.0 g/dl, T bilirubin (less than 1.2 mg/dl)), but direct bilirubin was slightly high (0.560 mg/dl) (normal value 0.0 to 0.25 mg/dl). Viral screening was negative, urinalysis showed uncountable pus cells. CRP (5.38 mg/l) (normal value 0.0 to 5.0) and ESR (50.0) (normal value <20 mm/h) were high. Blood grouping is A+.

**Fig. 1:**
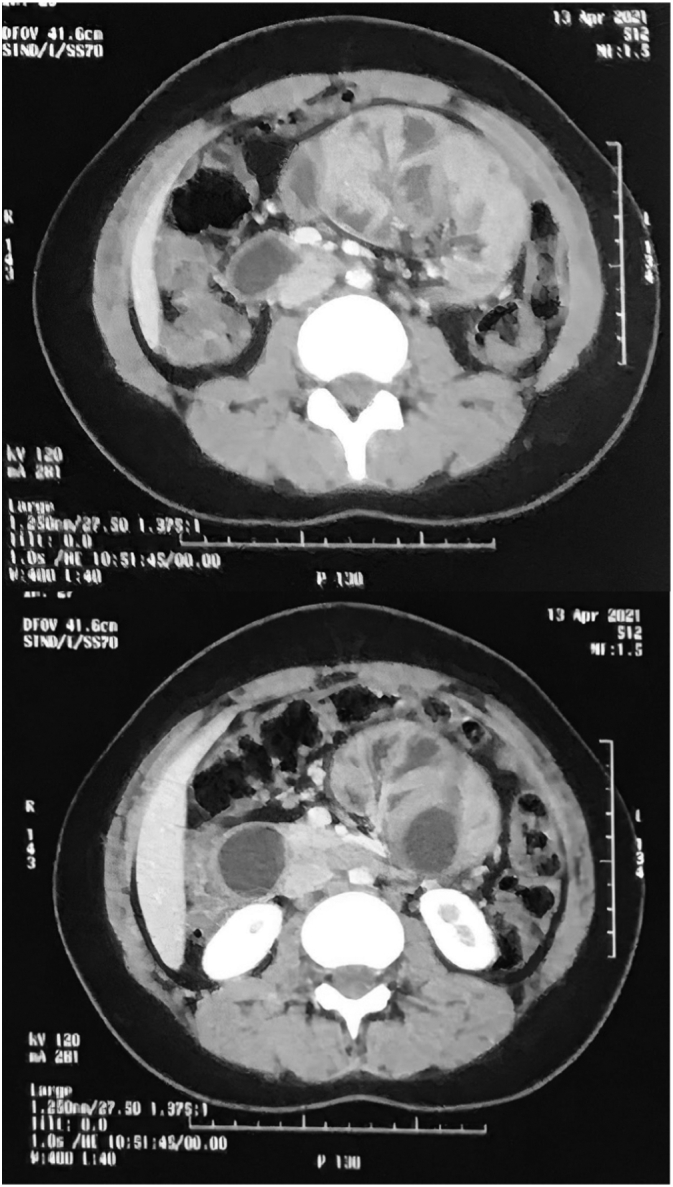
A CT scan showing dilated loops of small bowel.

Abdominal ultrasound showed a mildly enlarged liver with excessive bowel gases, but otherwise, it was unremarkable. CT scan of abdomen and pelvis (Fig. 1) shows a cluster of small bowel loops obstruction and mild dilation of proximal loops but does not confirm the diagnosis of PSEP a differential diagnosis of para duodenal hernia was suggested radiologically and excluded intraoperatively.

## 6 Intervention

**Fig. 2:**
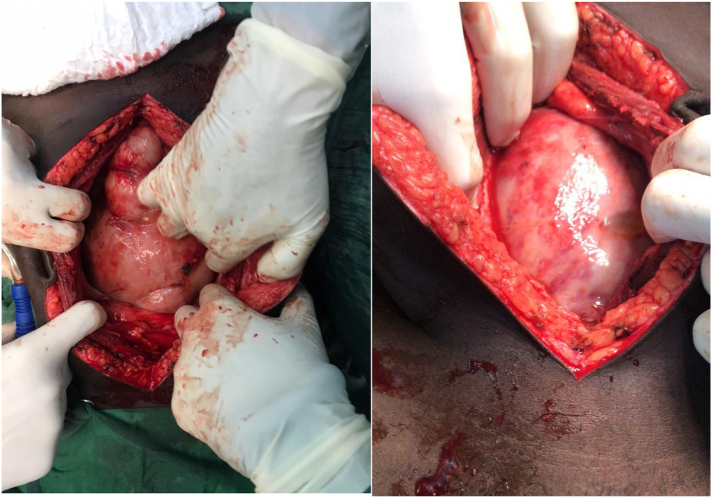
A laparotomy showing a thick fibrous membrane that resembles a cocoon.

**Fig. 3:**
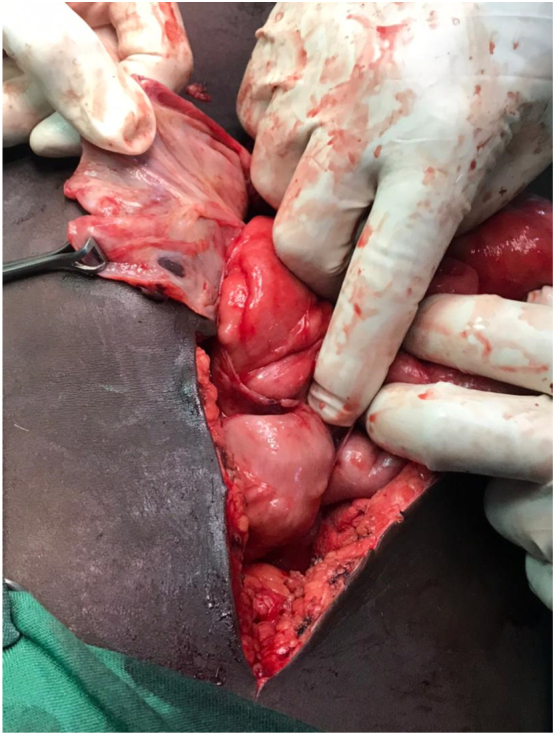
Releasing the fibrous membrane.

Based on the presence of abdominal pain, constipation, vomiting, and abdominal distension along with the radiological findings, a diagnosis of bowel obstruction was made, and the patient was managed conservatively (including NPO, analgesia, antibiotics, PPI, and supportive management). Three days later, a laparotomy was conducted due to the failure of the conservative management. Intraoperatively a shiny grey-whitish cocoon-like sac encapsulating the small bowel was observed (Fig. 2). The sac was opened and excised (Fig. 3), with adhesiolysis and appendectomy. Two lymph nodes were taken. All the excised and removed tissues were sent for histopathology. There was no intra-abdominal collection of fluid therefore no sample was taken for culture and sensitivity.

Operator: Mr. Nadir Salih, a senior consultant of general surgery. He attained his fellowship at the Royal College of Surgeons in Ireland in 2002. He progressed from surgical SHO to surgical registrar then to a senior surgical registrar, he did covers as a locum consultant in Ireland before he returned to Sudan in 2010. He joined the Faculty of Medicine, Alzaiem Alazhari University as an assistant professor, and as a consultant surgeon in Khartoum North Teaching Hospital in the same year. The operation was done in a private hospital in Khartoum state.

## 7 Follow-up and outcome

The patient was discharged seven days post-operatively with no in-hospital complications. Two weeks after discharge, and at the clinic for follow-up, there were no complications nor evidence of relapse. The histopathology report revealed mild chronic inflammatory infiltrate in the submucosa on the appendix. The serosa and peri-appendiceal adipose tissue showed congestion, edema, and infiltration by mixed inflammatory cellular infiltrate. There was no evidence of neoplasm or specific granuloma in the sample examined, which excluded abdominal tuberculosis. The features are consistent with those of peri-appendicitis. Microscopy of mesenteric lymph nodes showed normal architecture with marked follicular hyperplasia with expanded parafollicular T cell region. There was no evidence of granuloma or malignancy in the biopsy. The features are consistent with those of reactive follicular hyperplasia. Four months later, a second follow-up phone call revealed no recurrence of her symptoms, and she was leading a normal life.

## 8 Discussion

Primary sclerosing encapsulating peritonitis (PSEP) is a rare disease entity in which there is a thick, grayish-white membrane made of fibrous tissues and collagen enclosing the abdominal structures partially or totally, a cocoon-like sac [1].

There is no identifiable cause for Primary sclerosing encapsulating peritonitis (PSEP), although a role of cytokines, fibroblasts, and angiogenic factors has been suggested [4]. It classically presents in young females from tropical and subtropical countries [1]. A systematic review conducted in 2014 showed that while over 140 cases have been reported from China and India alone, only 6 cases have been reported from Africa including 5 cases from Nigeria and one case from Senegal [1].

More than half of patients have been diagnosed during the surgical operation and 16.7–48.7% of patients have their diagnosis preoperatively according to large case series conducted in China [5] and India [6].

While there is no absolute agreement on the best diagnostic and therapeutic approach as most of the studies reported on this disease are case reports with only one systematic review available, dated to 2015 [7], a contrast-enhanced CT scan seems to be the modality of choice for SEP [8]. The key findings that appear on the CT include matted dilated bowel segments with a thick peritoneal membrane (>2 mm), adhesions, which give the operating team an idea about the duration and difficulty of the surgery, and ascitic fluid between bowel loops [8] (also in the inguinal fossa [9]). Other findings reported include a Bottle Gourd sign (a dilated second and third part of the duodenum with encasement of the distal duodenum and proximal jejunum) [10], smudged appearance of the greater omentum [11], and focal or diffuse calcifications on the membrane or lymph nodes. Lymphadenopathy has been reported, mostly in cases of abdominal tuberculosis as a primary cause. The contrast-enhanced CT scan can also be of great value in detecting complications including bowel ischemia (or strangulation) manifested as lack of bowel enhancement as well as perforation. There are also scoring systems based on the CT findings but they are more useful for academic purposes because they do not correlate with the outcome [8].

Additionally, A clue to the diagnosis may be apparent on barium studies of the small bowel as a cauliflower sign [7], [12]. Abdominal X-rays may show a dilated loop of bowel with air-fluid levels, features consistent with bowel obstruction but not specific to SEP.

That is being mentioned, most cases, especially in young population, are diagnosed during laparotomies after the failure of conservative management of the bowel obstruction with a combination of clinical, radiological, surgical, and histopathological findings being the best way to have an ultimate and sure diagnosis given the rarity of this disease entity. Excision of the membrane and adhesiolysis, whether through an open approach or laparoscopically is the usual management.

There is a limited number of cases reported from the African continent when compared to other regions of the world, including 5 reported cases from Nigeria and one reported case from Senegal [13], [14], [15], [16] Patients ages ranged between 10 and 18 years and the majority (80%) of them were adolescent females with only one male case reported. This can support a theory postulating that the cause of such a condition might be retrograde menstruation [13]. We believe that the disease is underreported from this part of the world, given the fact that the disease is much more reported in other tropical and subtropical countries and a lot of cases are actually yet to be reported. An additional cause might be the difficulties that patients could face to reach hospitals especially in rural areas and have adequate surgical care. Therefore, to make sure more cases come to light, we recommend more careful consideration of unusual findings during exploratory laparotomies and to make surgical services available in rural areas and areas that have no or limited access to healthcare. That is being said, this can be difficult considering the fact that many of these African countries are actually limited-resource countries.

## Consent

Written informed consent was obtained from the patient's parent for publication of this case report and accompanying images. A copy of the written consent is available for review by the Editor-in-Chief of this journal on request.

## Provenance and peer review

Not commissioned, externally peer-reviewed.

## Ethical approval

Ethical approval was obtained from Ethical committee at Alzaiem Alazhari University.

## Funding

Authors received no funding from any individual or institution and this work is completely a voluntary work.

## Guarantor

Farah Mohammed.

## Research registration number

Not applicable.

## CRediT authorship contribution statement

1. Farah Mohammed: Involved in study design, data acquisition, drafting the article, revising it critically and finally approved the manuscript.
2. Mohamed Abdulkarim: Involved in study design, data acquisition, drafting the article, revising it critically and finally approved the manuscript.
3. Ammar Ibn Yasir: Involved in conception of the study design, drafting the article and finally approved the manuscript.
4. Osman Taleballah: Involved in conception of the study design, drafting the article and finally approved the manuscript.
5. Dafalla Shani: Involved in the design of the study, revising it critically and finally approved the manuscript.
6. Nadir Salih: Involved in the design of the study, revising it critically and finally approved the manuscript.

## Declaration of competing interest

Authors report no conflict of interest of any sort.
